# Postural Control and Gait Performance in the Diabetic Peripheral Neuropathy: A Systematic Review

**DOI:** 10.1155/2016/9305025

**Published:** 2016-07-20

**Authors:** Amirah Mustapa, Maria Justine, Nadia Mohd Mustafah, Nursuriati Jamil, Haidzir Manaf

**Affiliations:** ^1^School of Physiotherapy, Faculty of Health Sciences, Universiti Teknologi MARA, Puncak Alam Campus, 42300 Puncak Alam, Selangor, Malaysia; ^2^Discipline of Rehabilitation Medicine, Faculty of Medicine, Universiti Teknologi MARA, Sungai Buloh Campus, 47000 Sungai Buloh, Selangor, Malaysia; ^3^Digital Image, Audio & Speech Technology Research Group, Faculty of Computer and Mathematical Sciences, Universiti Teknologi MARA, 40450 Shah Alam, Selangor, Malaysia

## Abstract

*Purpose*. The aim of this paper is to review the published studies on the characteristics of impairments in the postural control and gait performance in diabetic peripheral neuropathy (DPN).* Methods*. A review was performed by obtaining publication of all papers reporting on the postural control and gait performance in DPN from Google Scholar, Ovid, SAGE, Springerlink, Science Direct (SD), EBSCO Discovery Service, and Web of Science databases. The keywords used for searching were “postural control,” “balance,” “gait performance,” “diabetes mellitus,” and “diabetic peripheral neuropathy.”* Results*. Total of 4,337 studies were hit in the search. 1,524 studies were screened on their titles and citations. Then, 79 studies were screened on their abstract. Only 38 studies were eligible to be selected: 17 studies on postural control and 21 studies on the gait performance. Most previous researches were found to have strong evidence of postural control impairments and noticeable gait deficits in DPN. Deterioration of somatosensory, visual, and vestibular systems with the pathologic condition of diabetes on cognitive impairment causes further instability of postural and gait performance in DPN.* Conclusions*. Postural instability and gait imbalance in DPN may contribute to high risk of fall incidence, especially in the geriatric population. Thus, further works are crucial to highlight this fact in the hospital based and community adults.

## 1. Introduction

Diabetic peripheral neuropathy (DPN) is a debilitating microvascular complication among the patients with Type 2 Diabetes Mellitus (T2DM). Approximately 49.3% of T2DM patients have DPN in Iran [1]. A study in Sri Lanka found 48.1% of DPN diagnosed using Diabetic Neuropathy Symptom (DNS) score among 528 diabetic patients [2]. A study in India reported that every fifth individual diagnosed with T2DM is likely to have DPN [3]. In Malaysia, about 14.4% of 215 T2DM patients in Kelantan were reported to have DPN [4]. These figures are alarming as the local prevalence of diabetes is increasing from 11.6% in the year 2006 [5] to 15.2% in the year 2011 [6] due to the aging of the population.

DPN is explained by the presence of signs and symptoms of peripheral nerve dysfunction in diabetic patients after nondiabetes causes have been excluded [7]. DPN affects the proximal and distal peripheral sensory and motor nerves [8, 9]. Sensory neuropathy is prominent in DPN with an exhibition of numbness and prickling sensation in a stocking-glove pattern that starts from the feet and spreads proximally [10]. DPN also affects the autonomic nervous system, known as diabetic autonomic neuropathy [8, 9, 11] with manifestation on the exercise intolerance, orthostatic hypotension, and sudden death [11].

The most common symptoms of DPN are numbness, tingling, and pain that may worsen during the evening. It begins in the toes towards plantar of the feet, ankles, and lower shins with the association of night cramps [7] and unsteadiness in walking [3]. Gradually DPN will affect distal muscle strength and deteriorates normal walking function [10]. Alteration of the peripheral nerve due to DPN is the chief contributor to postural instability [12] and high gait variability that may increase the likelihood of fall incidence [13].

Individuals with DPN are 15 times more likely to experience fall compared to the healthy subjects [14]. Falls are marked as a dangerous health issue in DPN especially in the geriatric population [15]. Therefore, knowledge of the factors that influence falls such as postural control deficit and gait instability in DPN patients is essential. Thus, the aim of this paper is to give an in-depth review of the published studies on the characteristics of impairments in the postural control and gait performance in DPN.

## 2. Methods

### 2.1. Search Strategy

To provide a comprehensive review of postural control and gait performance in DPN, an electronic search was performed within the Google Scholar, Ovid, SAGE, Springerlink, Science Direct (SD), EBSCO Discovery Service, and Web of Science databases until April 1, 2016. The keywords used for literature search were “postural control,” “balance,” “gait performance,” “diabetes mellitus,” and “diabetic peripheral neuropathy.” The searching was limited to English, academic journal, and human studies only.

### 2.2. Study Selection

All identified titles and citations retrieved by the search strategy were screened to select relevant studies. Two reviewers independently checked the titles and abstracts of all the selected articles. In discrepancy case between the reviewers, a consensus meeting was done to make a final decision on the articles selection. Then, the selection criteria were applied to all potentially relevant full text of articles. Articles were eligible if they met the following inclusion criteria:

1. The studies and reports that provided data on the postural control and gait performance in DPN.
2. The study that indicated cross-sectional and case-control (narrative review and single case studies were excluded).
3. The study population, that is, patients with diabetic neuropathy or DPN (studies on patients with diabetes mellitus without identified into DPN were excluded).

### 2.3. Data Extraction

The full text of the articles was gathered and reviewed if the study was appropriate. Data on the study characteristics (study design, author, and year), population (age, gender, and number), the methodology used, and the characteristics of postural control and gait performance in DPN were extracted. The PRISMA guidelines for reporting literature searching were utilized in this study [16].

## 3. Results and Discussion

**Figure 1:**
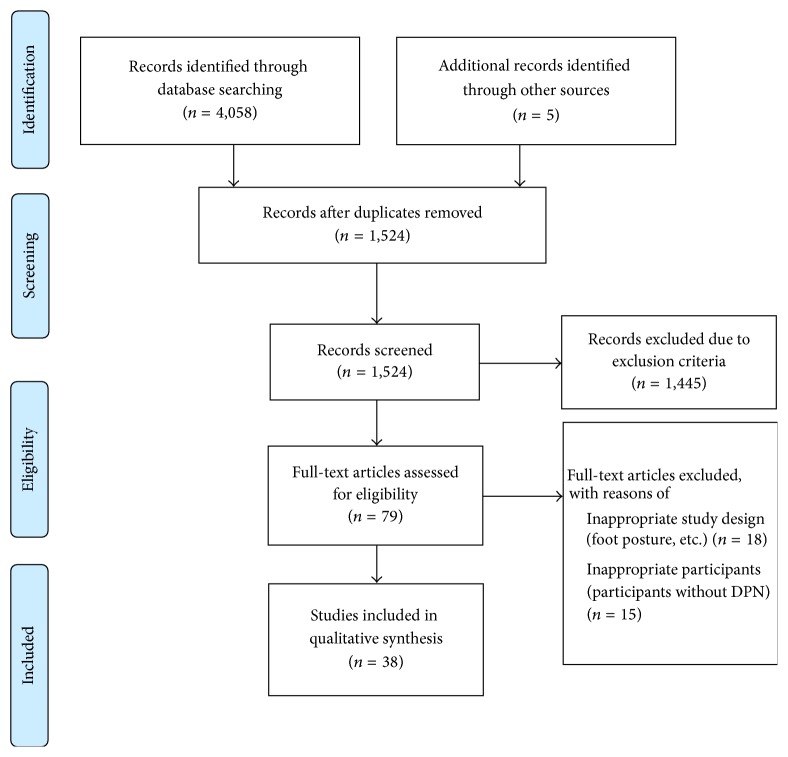
Flowchart of studies selection.

4,337 studies were hit in the search (Figure 1). 1,524 studies were screened on their titles and citations. Then, 79 studies were screened on their abstract. Only 38 studies were eligible to be selected: 17 studies on postural control and 21 studies on the gait performance. Figure 1 displays the process of selecting the studies.

### 3.1. Study Characteristics

Tables 1 and 2 show the characteristics of selected studies.

### 3.2. Participants Characteristics

This study reviewed 883 DPN participants and various methods were used to identify DPN among the participants. Some studies evaluated DPN by recording nerve conduction velocity (NCV) [17, 21, 27, 29] through amplitude and latency tests of sensory (sural) and motor (peroneal and tibial) nerves [27, 38]. Additionally, the Michigan Neuropathy Screening Instrument (MNSI) test with score higher than eight [22, 35, 45] included a test of vibration sensitivity using a tuning fork set at 128 Hz; tendon reflexes, muscle strength in the lower extremities [20, 50], and the somatosensory test using Semmes-Weinstein monofilaments examination (SMWE) were used to diagnose DPN [19, 28, 37, 38, 49, 53, 54]. The presence of DPN also was assessed by using the modified Neuropathy Disability Score (mNDS) NDS > 5 [42, 48] and the vibration perception threshold (VPT) [36] with <25 volts [24, 29, 30, 34, 40, 46, 55]. mDNS consists of clinical testing of (1) sensory modalities (pinprick, light touch, vibration, and pain), (2) the anatomic level below which light touch sensation is impaired, (3) muscle strength, and (4) ankle jerk. The total score varies between 0 and 33. A total score of 0 is graded as no DPN, 1–9 as mild, 10–18 as moderate, and 19–33 as severe DPN [18].

### 3.3. Instruments and Methods Used for Postural Control Assessment

Three studies used static posturography on a standard platform [20, 24] and a GS-10 Anima gravicorder (Anima Corporation, Tokyo, Japan) to assess postural control [33]. Additionally, the Physilog system (BioAGM, CH) was used to measure linear accelerations at the trunk and ankle levels of postural control [31]. A study used dual force platform with a touch plate, positioned at the participants' right side at 90 cm height from the floor and examined under three touch conditions, no touch (NT), light touch (LT), and heavy touch (HT) to measure postural control [19]. Dynamometric platform (Kistler) also was used to calculate sway area in EO and EC, while platform producing a horizontal sinusoidal in the AP direction, alternately with EO and EC, was used to measure dynamic balance [25]. Similarly, Sliding Linear Investigative Platform, a horizontal translating force platform [23], and standing on foam rubber mat (40407.5 cm thick) in EO and EC [54] were used to assess postural sway. A recent study that used traditional body sway parameters from COG plots includes COG (AP) sway, COG (ML) sway, and COG sway with two 15-second trials of stood upright with feet together as close as possible and with arms crossed over chest in EO and EC [30]. Force platform measurements (Kistler) [17, 18, 27, 29] and Wii Balance Board® [26] were used to evaluate the COP fluctuations to identify postural sway. Some studies also evaluate COP through a static balance test carried out in EO and EC conditions [17, 26].

### 3.4. Instruments and Methods Used for Gait Performance Assessment

Portable electronic GAITRite walkway system [40] and electronic baropodometry treadmill (FootWalk Pro AM CUBE, France) with a sampling frequency of 200 Hz, equipped with the software FootWork Pro [45], were used to calculate spatiotemporal gait parameters, while other studies assessed gait by walking along an 11 m pathway called the Walk Ratio at preferred and maximum speeds to measure cadence [37]. Walking speed was assessed by measuring the time taken to walk a 10-metre distance following a 3-metre predistance to assure constant velocity [56] and equipped with the Physilog1 system to walk with a preferred walking speed [34]. A study used gait analyzer system (LEGSys BioSensics LLC, Cambridge, Massachusetts) wearable sensors attached to the right and left anterior shins, the right and left anterior thighs, and posteriorly to the lower back for gait analysis [46]. Another study asked participants to walk on a motorized treadmill at a constant speed of 1 m/s and filmed them with a 60 Hz video camera and automatic digitizing software (Peak Performance, Englewood, CO) for gait analysis [14]. One study performed gait analysis by using a nonslippery steel-covered pathway 8 m long with the conductive material under the heel and toes of each foot to provide accurate temporal values corresponding to the onset and offset of right and left single support and double support phases [39]. Gait analysis was performed by using a 6.7 m long pressure sensitive carpet (GAITRite1, CIR System, Havertown, USA) with three different locomotion speeds slow speed, preferred speed, and maximal speed in EO and during preferred walking in EC [51] and by wearing flat-soled athletic shoes (New Balance Athletic Shoe Inc., Boston, MA) [35].

Gait stability was analyzed with a baropodometer (FootWalk Pro, AM CUBE, France; sampling rate of 200 Hz) consisting of a 2 m pressure platform and a 6 m walkway (total of 8 m), which permits gait acceleration and deceleration in the initial and final 3 m walked on the walkway at a comfortable and self-selected speed in conditions EO and EC and walking with EO and narrow base of support (NB) [41], while kinematic and kinetic data were acquired by using (1) single camera motion capture [44], (2) a BTS motion capture system (six cameras, 60–120 Hz) synchronized with two Bertec force plates (FP4060-10) and integrated with two Imago plantar pressure systems (0.64 cm^2^ resolution, 150 Hz) [28], (3) a Vicon 512 Motion Analysis System (Oxford Metrics Ltd., Oxford, England) with six cameras operating at a sampling frequency of 100 Hz and a force plate (Kistler, Switzerland) embedded into a 10 m walkway operating at a sampling frequency of 400 Hz [48], (4) a portable data logger based on Tattletale Model 8 hardware (Onset Computer, Inc., Pocasset, MA), which consisted of a fully programmable microprocessor, an 8-channel 12-bit A/D converter [13], (5) GaitMeter*™*, a portable and inertia sensing motion analysis system attached to the anterior mid shins [53], and (6) 2D digital optical recording system, which consisted of a 25 Hz interlaced digital video camera (50 frames/s) (MX5, Adimec, Holland) with a 12.5 mm lens (Ernitec) [50]. A study used the Vifor (video force) system to identify patterns of gait and kinetic data in the sagittal and frontal planes during walking [42].

### 3.5. Characteristics of Postural Control Impairments in the DPN

Postural control is defined as the control of body's position in space for balance purpose. Postural control is obtained from sensory feedbacks of the body which are the vestibular, visual, and somatosensory system [57]. Postural control in static conditions is known as postural steadiness while in the dynamic volitional perturbations it is noted as postural stability [58]. Postural instability was significantly associated with DPN compared to the DM only and control group through imbalance of standing in two studies [29, 33]. But, Simoneau et al. (1994) further tested the postural stability under EO/head straight, EO/head back, EC/head straight, and EC/head back condition [29]. de Souza Fortaleza et al. [22] reported a significant difference in the Mean Amplitude Oscillation (MAO) A/P EO and EC, MAO-M/L EC and Semitandem (ST), and Average Speed Oscillation (ASO), ASO-M/L ST, were identified as the factor of postural instability among DPN group [22]. A study by Fulk et al. (2010) revealed diabetic with and without DPN requires accelerations to detect a 1 mm and 4 mm displacement, and DPN may not be the only cause of impaired balance in people with DM [23]. Turcot et al. (2009) revealed significantly higher anterior-posterior range of lumbar acceleration and ankle accelerations for DPN group compared with other groups, interpreting greater postural instability. This study also suggested visual deficit as contributing factors of greater postural instability in DPN [31]. Supportingly, several studies also supported that the absence of visual and vestibular senses lead to worsening of posture control [18, 21, 22, 29].

Postural instability in DPN occurs due to deficits of systems that work to control balance. Reducing accurate feedback of the proprioception sense along with the deterioration of somatosensory [21], visual [18, 21, 22, 29], and vestibular systems causes postural instability and larger postural sway [21]. Interruption of the afferent and efferent neuron function through the termination of the tibial, sural, and deep peroneal nerves in the mechanoreceptors of the capsule and ligaments at the ankle joint [59] lead to diminishing function of proprioceptive and tactile sensation in maintaining postural stability [12]. However, review by Bonnet and Lepeut (2011) questioned whether peripheral neuropathy may exaggerate postural control mechanisms as they found contrast result from five studies regarding the issues of neuropathy problem in disruption of postural control [60]. Visual dysfunction in DM occurs when peripheral vision is occluded with high blood glucose level in the blood vessels of the retina [61]. DM affect vestibular function as the vestibular system is sensitive to high blood glucose and insulin level, which cause DPN group exhibited impaired ability to detect short, whole body anterior translation with large sway area [62].

Postural sway is defined as the response of the postural muscle activity in stimulating a continuous to-and-fro movement of the body against the point of gravity while standing [32]. Dixit et al. (2015) [20] found a significant difference with greater sway amplitude on firm and foam surface in EO, EC, and EO on foam and EC on foam conditions in DPN group. Fahmy et al. (2014) also demonstrated that DPN group had significantly lower equilibrium scores, lower balance score of the Berg Balance Scale (BBS), and more postural sway than the control group [21]. One study noted that trace surfaces were significantly larger; trace length was significantly longer and the mean velocity of body sway (MVEL) was faster in EO and EC in the DPN group than the other groups [32], while another study demonstrated the velocity of body sway and velocity variance and the anteroposterior mean position of the body (VFY), fast Fourier transformation on *x* (FFTX), and fast Fourier transformation on *y* (FFTY) planes frequencies of body oscillation on *x* and *y* planes were significantly higher in DPN group compared to the DM without DPN and control group [24]. Body sway (COG (AP) sway, COG (ML) sway, and COG sway) in EO and EC in DPN group was significantly higher by 74% with EO and 87% with EC than controls [30]. Boucher et al. (1995) found significant postural sway through investigating five postural dependent variables (anteroposterior, mediolateral, and scalar ranges of sway, sway speed, and dispersion of sway) in DPN group than the control group [17]. Decrease in postural sway at low-medium frequencies suggested lower reliance on vestibular system. Also, this study noted that DPN group showed better balance and sway compensation performance after balance training [27]. One study concluded that DPN group had a significantly larger Centre of Pressure-Centre of Mass (COP-COM) amplitudes in the eyes closed (EC) condition compared with the eyes open (EO) condition in anteroposterior (A/P) and mediolateral (M/L) directions than the control group [18]. A study by Dickstein et al. (2003) noted significant longer postural response latencies and smaller scaling of initial response magnitude in DPN group compared to the control group [19]. Normal adult maintains their posture by an anterior-posterior sway pattern which is known as ankle strategy, resembling an inverted pendulum: the fulcrum is the ankle, and the head is the opposite end of the pendulum. However, DPN group exhibited reduced ability of the ankle strategy to resist postural sway against gravity as proven by the finding of larger trace surface of posturography in DPN group compared to the control and diabetic without DPN group [32]. Consistently, postural sway in DPN occurs following the inability of the postural and lower limb muscles to provide an adequate activity level of muscles and joint. Muscle strength reduction is associated with relatively high glucose level and potentially less glucose uptake and hyperglycemia in muscles, which can contribute to lower capability in resisting postural sway [17, 30]. Abnormality of the postural sway is recognized and expected if there is any deterioration of one sensory input and/or motor output. Evidently, postural sway requires high demands on the peripheral nervous system. Therefore, impairments of the afferent and the efferent pathways in DPN are suggested to cause many considerable changes in postural sway reaction [32]. In spite of that, review by Bonnet and Ray (2011) partially agreed with findings of DPN being the fundamental reason causing larger postural sway in diabetic patients than controls in quiet stance and affecting more if visual or vestibular systems are deteriorated [63].

For static balance alteration, a study by Palma et al. (2013) revealed that DPN group showed poor balance under both EO and EC conditions, with a significantly greater COP ratio in the CE condition [26]. Nardone et al. (2006) also concluded that DPN group are unstable during quiet stance compared to the control group with slight increase of the head AP displacement despite displacement of the feet during dynamic balance task [25]. In addition, DPN also demonstrated significant high reaction time and reduce movement velocity, which noted slow sensory processing and motor planning deficits [21]. Indeed, DPN affects somatosensory input that is a proprioceptive and tactile sensation and motor output, that is, reaction time and muscle strength that contribute to abnormal postural control [23].

### 3.6. Characteristics of Gait Performance in the DPN

Temporal-spatial parameters were significantly affected in DPN group [34, 37]. There were smaller step length [39, 45, 47, 49, 54], reduced duration of single support [39, 47], higher duration of double support [45, 47, 51], decreased gait velocity [38, 42, 47, 49, 51], lowered cadence [47, 54], increased step width-to-step length ratio [49], increased step time and step time variability [43, 49], and greater gait variability [51, 54] in the DPN group compared to the control group. DPN group had a significant reduction in gait speed and single stance time and increased double support time in experimental of walking with EO, EC, and EO and narrow base of support (BOS) compared to the control group [41]. The changes in this gait parameter were concluded as the factors of gait instability. Furthermore, DPN group significantly walked slower with shorter steps than control that causes increase in gait variability [13, 44].

Katoulis et al. (1997) demonstrated that alteration of gait parameters during walking among DPN group is a sequence of smaller maximum knee joint angle in the sagittal plane, higher maximum value of the anteroposterior forces, and higher maximum frontal plane ankle joint moment in DPN group compared to the normal group [42]. In addition, lower range of motion at the hips (frontal plane, by 25%), hips and knees (transverse plane, 31% and 32%), ankles (sagittal plane, 22%), and first metatarsophalangeal joints (sagittal plane, 32%), with less foot rotation (24%) identified gait alterations in people with clinically severe peripheral neuropathy and related plantar foot ulcer history [48]. The maximum value of the vertical component of GRF was found to be higher (*P* < 0.03) in the two control groups compared with the DPN group [42].

Brown et al. (2014) also reported that maximum joint strengths of the knee were significantly less in both diabetic with and diabetic without DPN and less strength of ankle in DPN group compared to the control group. This author suggested that reduced strength of knee and ankle may cause a disturbance in perturbation response in balance, potentially increasing the risk of falling [36]. Additionally, Sawacha et al. (2009) noted that trunk and lower limb joint mobility (in static and dynamic states) were more reduced in diabetics with and without DPN in each plane with DPN significantly demonstrating lower ranges of motion. Furthermore, both diabetic only and DPN groups showed significant reductions in each joint moment and velocity during gait [28]. Gait variability and the coefficient of variation of gait velocity were significantly higher in the DPN group when barefoot walking over long distances. Furthermore, there was a high correlation between neuropathy severity and gait unsteadiness demonstrated during the barefoot walking/long walking distance condition [46]. DPN with a history of fall demonstrated decreased speed, greater step width (SW), shorter step length (SL), and greater SW-to-SL ratio (SW : SL) on both surfaces compared to the DPN with no history of fall [42].

Gait in DPN is known as a conservative gait performance that occurs with high double support time, slow speed, and shorter steps as an attempt to keep stability in walking. This pattern is exhibited by DPN because of the reduction of proprioception sensory feedbacks from the lower extremities [39] and weakness of ankle plantar flexor and dorsiflexor muscles [41, 47, 49, 54]. Less dorsiflexion mobility and increased plantarflexion mobility were associated with a decrease in muscle strength of the dorsiflexors and plantiflexors, which may affect speed of the gait cycle [45]. Moreover, the improper input of vestibular and visual systems also cause CNS to lose coordination in gait and may cause decreased gait speed and wider step length [51, 64]. Moreover, lack of sensation towards pain and pressure with repetition of high pressure on the forefoot during the push-off phase of gait may result in inflammation and then develop ulceration among neuropathies [65]. Consequently, foot pain may cause antalgic gait with smaller steps in DPN patients [66]. In fact, diminished sensory feedback with a further contribution of vision impairments, muscle weakness, and lack of neuromuscular control of distal joints in neuropathic patients results in increase of gait instability [54].

Additionally, diminished sensory information makes gait control more cognitively dependent in diabetic neuropathic persons than in control subject [39], due to the inability of the brain to respond on the dual tasking in walking [47]. Type 2 DM is associated with the dysregulation of glycemic variability that might contribute to brain atrophy and cognitive impairments [67]. Diabetes significantly causes impairment in information processing speed, memory, and attention with attribute effect on the mood states [68]. Furthermore, individuals with diabetic retinopathy have shown reduced performance on cognitive tests of fluid intelligence, information processing speed, and ability to maintain attention and concentration [69]. de Mettelinge et al. (2013) found older adults with diabetes with impaired cognitive function walked slower, took shorter strides, and had reduced double support time and increased gait variability compared with participants with intact cognitive function. Therefore, gait was further affected by reduced cognitive function, irrespective of the presence of neuropathy [40]. Cognitive and attention impairments have an important role in the maintenance of balance and postural control as the brain gives the command to the limb muscles to stabilize the body [47]. Thus, diabetic patients with cognitive and attention impairments will have balance deficit in walking and lead to a high risk of fall incidence. Relatively, study by Pan and Bai (2014) has reviewed appropriate balance training including proprioception, ankle strategy, and vestibular training as an intervention to reduce fall risk among elderly with DPN [70].

## 4. Conclusion

This systematic review evaluated 38 studies, investigating characteristics of postural control and gait performance impairments in 883 DPN participants. A huge number of articles published about this study reflect the importance of investigations and researches in this aspect. Pandemic prevalence of DPN and the well-known fall incidence require understanding of the postural control and gait performance changes which DPN patients are confronted with. Overall, this review has made an endeavor to state two points. First, DPN patients have been demonstrated with postural instability and gait imbalance that contribute to fall incidence. Lastly, DPN patients exhibited significant deficit in sensorimotor function, balance, and gait. Cognitive and attention impairments also have an important role in maintaining balance in gait performance as the brain gives the command to the limbs muscles to stabilize the body. Therefore, cognitive impairment will result in delay or slow response of dual tasking action. Consequently, this condition may lead to many deleterious effects if individuals with cognitive impairment do dual tasking in mobility such as walking or turning. Holistic managements are required to cope and prevent this problem. In particular, the practice of physiotherapy requires theory-based knowledge and evidence-based practice. Thus, further works in this aspect are crucial to providing evidence to support the need for balance and gait training that can be used by physiotherapists in the clinical practice as one of the primary strategies of rehabilitation in DPN patients.

## Figures and Tables

**Table 1:** Study characteristics on the impairments of postural control's studies.

Author	Sample size	Age	Groups	Examined variables	Procedure	Results and conclusion
Boucher et al. (1995) [17]	29	Not stated	DPN (17) Control (12)	Postural sway: (1) sway range; (2) sway speed; (3) sway dispersion	Kistler piezoelectric force platform was used to measure the displacement of the COP foot in EO and EC conditions.	DPN showed a larger range of sway, faster sway speed, and greater sway dispersion than control in EO and EC.

Corriveau et al. (2000) [18]	30	>60	DPN (15) Control (15)	COP-COM in A/P and M/L	Standing on two adjacent force platforms in EO and EC condition.	DPN group demonstrated less stable posture than the control group, with EO and EC.

Dickstein et al. (2003) [19]	18	Not stated	DPN (8) Control (10)	Postural responds	Standing barefoot with EC on the surface of a dual force platform. Each subject was tested under three touch conditions—no touch (NT), light touch (LT), and heavy touch (HT)—during three backward translation velocities of 10, 20, and 30 cm/s.	Postural response latencies were significantly longer and scaling of initial response magnitude in proportion to translation velocity was significantly smaller in the DPN group compared to the control group.

Dixit et al. (2015) [20]	61	Not stated	DPN (61)	Postural stability	Force platform in static stance and at pelvis width in EO, EC, and EO on foam and EC on foam.	There was a significant difference in DPN group, with greater sway amplitude on firm and foam surface in all the conditions.

Fahmy et al. (2014) [21]	60	40–50	DPN (30) Control (30)	Postural stability (effect of proprioceptive sense and vestibular function)	BMS force plate: (i) LOS test: maximum distance a person can displace his COG without losing balance. (ii) mCTSIB: standing on the force plate with EO and then EC, using firm and foam surfaces successively.	DPN group reduced postural stability in EC compared to the control group. Affection of foot sensation and proprioception in DPN may affect the result of LOS and postural stability examination.

de Souza Fortaleza et al. (2013) [22]	30	55–70	DPN (13) Control (17)	Postural control: (MAO-A/P, MAO-M/L, ASO-A/P, and ASO-M/L)	MAO and ASO were analyzed by a kinematics system with standing position for 30 seconds in EO, EC, and ST.	There was increase in MAO-A/P (due to vision) and MAO-M/L (due to reducing BOS) in DPN in the EO and EC. MAO-A/P failed to show changes in the ST position in the DPN group.

Fulk et al. (2010) [23]	83	Not stated	DM with PN (25) DM without DPN (7) DPN without DM (19) Control (32)	Postural perturbation	Sliding Linear Investigative Platform for Assessing Lower Limb Stability (SLIP-FALLS).	DM with and without DPN group required higher accelerations to detect a 1 mm and 4 mm displacement. DPN may not be the only cause of impaired balance in people with DM.

Giacomini et al. (1996) [24]	54	Not stated	DPN (23) DM without DPN (10) Control (21)	Body sway: (1) velocity of COG sway; (2) VFY	Static posturography with EO and EC.	Mean velocity of sway, velocity, and VFY were higher in DPN group, which lead to instability.

Nardone et al. (2006) [25]	47	43–77	DPN (27) Control (20)	Reflex responses to stance perturbation (static and dynamic)	Static standing EO and EC on a tilting platform. Stabilometry (dynamometric platform). Dynamic balance (horizontal sinusoidal platform) in the A-P direction.	DPN group are unstable during quiet stance compared to the control group. During a dynamic balance task, the head AP displacement was only slightly increased in the patient groups with respect to normal group.

Palma et al. (2013) [26]	20	40–54	DPN (10) DM without DPN (10)	Static balance	Static balance was evaluated using the COP mean ratio on a Wii Balance Board® under EO and EC.	DPN group demonstrated worse static balance than participants without DPN in the EC.

Salsabili et al. (2013) [27]	19	40–70	DPN (19)	COP fluctuations AP and ML	Force platform.	DPN group has decreased in postural sway at low-medium frequencies showing lower reliance on vestibular system.

Sawacha et al. (2009) [28]	67	Not stated	DPN (26) DM without DPN (21) Control (20)	Posture	Plantar pressure systems.	Altered posture was found in diabetic patients irrespective of polyneuropathy.

Simoneau et al. (1994) [29]	51	40–70	DPN (17) DM without DPN (17) Control (17)	Postural stability	Standing on force platform under four conditions: EO/head straight, EO/head back, EC/head straight, and EC/head back.	DPN group had a significant effect on stability during standing, but DM and control groups had no effect.

Toosizadeh et al. (2015) [30]	36	>55	DPN (18) Control (18)	Body sway parameters	Standing with EO and EC with 2 sensors (triaxial accelerometer and triaxial gyroscope) attached to the ankle and hip joints.	Body sway (COG (AP) sway, COG (ML) sway, and COG sway) in EO and EC in DPN group was higher than that in the control group.

Turcot et al. (2009) [31]	36	Not stated	DM without DPN (12) DPN (12) Control (12)	Balance stability	Quiet standing balance was investigated using an accelerometric-based method in EO and EC. Accelerations were measured at lumbar and ankle levels using three accelerometers.	DPN group have greater postural instability with higher acceleration values than those of control group and DM without DPN.

Uccioli et al. (1995) [32]	54	Not stated	DPN (23) DM without DPN (10) Control (21)	Body sway: (1) trace length; (2) trace surface; (3) MVEL	Static posturography with EO and EC	There were larger trace surfaces, longer trace length, and faster MVEL in EO and EC condition in DPN compared to other groups. DPN group demonstrate high postural sway.

Yamamoto et al. (2001) [33]	110	Not stated	DPN (32) DM without DPN (23) Control (55)	Postural sway	Posturography	DPN group exhibited an inability to maintain an upright posture.

EO: eyes open, EC: eyes closed, BMS: Balance Master System, LOS: limits of stability, mCTSIB: Modified Clinical Test of Sensory Interaction on Balance, COP: Centre of Pressure, COM: Centre of Mass, COG: Centre of Gravity, A/P: anterior/posterior, M/L: medial/lateral, MAO: Mean Amplitude Oscillation, ASO: Average Speed Oscillation, ST: Semitandem, MVEL: mean velocity of body sway, and VFY: physiological ankle control to hip postural control.

**Table 2:** Study characteristics on the impairments of gait performance's studies.

Author	Sample size	Age	Groups	Examined variables	Procedure	Results and conclusion
Allet et al. (2009) [34]	45	Not stated	DPN (15) DM without DPN (15) Control (15)	Temporal and spatial gait parameters Stride-to-stride variability	Gait is assessed on three different surfaces (tar, grass, and stones) with a Physilog1 system (BioAGM, CH), consisting of accelerometers and gyroscopes.	Gait parameters of DPN were differed significantly from healthy controls. Post hoc analysis revealed a significant difference between healthy individuals and patients with neuropathy and between healthy individuals and patients without neuropathy.

Allet et al. (2012) [35]	33	50–85	DPN (21) Control (12)	Gait analysis: (i) gait speed and efficiency (step-width-to-step-length ratio)	An optoelectronic camera system measured kinematic data.	Hip adduction rate torque development (RTD) and ankle inversion RTD predicted 54% of gait speed, with the former predicting the majority (44%). Ankle inversion RTD was the only significant predictor of gait efficiency, which accounted for 46% of its variability.

Brown et al. (2014) [36]	80	Not stated	DPN (20) DM without DPN (33) Control (27)	Kinematics Kinetics	VICON (motion analysis).	DM and DPN group showed significantly reduced peak torques at the ankle and knee.

Camargo et al. (2015) [37]	60	Not stated	DPN (30) Control (30)	Temporal and spatial gait parameters	Measuring the time to walk a set distance during self-selected and maximal walking speeds.	Temporal-spatial gait, functional mobility, balance performance, and ankle muscle strength were affected in DPN group.

Chiles et al. (2014) [38]	983	>65	DM with and without DPN (126) Impaired fasting glucose (107) Control (750)	Gait speed	Short Physical Performance Battery (SPPB) by usual walking speed test (m/s).	DPN group showed lower walking speed.

Courtemanche et al. (1996) [39]	19		DPN (12) Control (7)	Gait	Walking task was performed on a nonslippery steel-covered pathway 8 m long.	For the walking task, DPN group had a smaller cycle amplitude, cycle speed, and single support time compared to the control group. Also, reaction times while walking were higher for DPN group than for control subjects. The increased attentional demands in gait for the DPN group, along with their more conservative gait pattern, suggest that a lack of proprioception from the legs affects the control of gait.

Dingwell et al. (1999) [14]	51	40–70	DPN (17) DM without DPN (17) Control (17)	Kinematic	Subjects walked on a motorized treadmill at a constant speed of 1 m/s.	DPN group did not demonstrate significantly greater variability than other groups.

de Mettelinge et al. (2013) [40]	101	>60	DPN (28) DM without DPN (28) Control (45)	Temporal and spatial gait parameters	Portable electronic GAITRite walkway system.	Compared with controls, older adults with diabetes walked slower, took shorter strides during all walking conditions, and showed more gait variability.

Fortaleza et al. (2014) [41]	41	Not stated	DPN (18) Control (23)	Gait stability: (1) gait speed; (2) percentage of time in double and single stance	Analyzed with an aropodometer by walking with EO, EC, EO, and narrow BOS.	DPN group showed lower gait speed, longer double stance time, and shorter single stance time in the three conditions.

Katoulis et al. (1997) [42]	60		DPN (20) DM without DPN (20) Control (20)	Gait parameters: (1) walking speed; (2) stance phase duration; (3) joint angles and moment arms for the ankle, knee, and hip joints in both sagittal and frontal planes; (4) the components of the ground reaction force (GRF) vector; (5) the ankle, knee, and hip joint moments originating from the GRF vector in both planes	Vifor (video force) system (LIC Orthopaedics, Stockholm, Sweden).	Walking speed was significantly slower in the DN group compared with the two control groups. The maximum knee joint angle was smaller in the sagittal plane for the DPN group compared with the control group values. The maximum value of the vertical component of GRF was found to be higher in the two control groups compared with the DPN group. The maximum value of the anteroposterior forces was also found to be higher in the control group compared with the DPN group.

Lalli et al. (2013) [43]	86	Not stated	DPN without pain (20) DPN with pain (22) DM (20) Control (24)	Gait parameters (variability of step length and step velocity)	Collection of GaitMeter data was performed during a single 10–20-minute session.	DPN group had greater variability of step length and step velocity, except for DM and control group. DPN group with pain contributes to gait variability, potentially contributing to the risk of falling in DM patients.

Manor et al. (2008) [44]	24	Not stated	DPN (12) Control (12)	Kinematic Gait variability	Two-dimensional sagittal plane kinematics were acquired (60 Hz) using single camera motion capture.	DPN group walked slower than the control group.

Martinelli et al. (2013) [45]	52	Not stated	DPN (25) Control (27)	Temporal and spatial gait parameters	Electronic baropodometry treadmill with walk on the treadmill (8.0 m) at her/his habitual self-selected speed.	DPN group showed impairment of gait, with a smaller stride and length speed of the cycle, and increased the duration of support time.

Najafi et al. (2013) [46]	20	>18	DPN (10) Control (10)	Temporal and spatial gait parameters	A validated system of body-worn sensors was used to extract spatiotemporal gait parameters.	Gait alteration in DPN group is most pronounced while walking barefoot over longer distances and that footwear may improve gait steadiness in patients with DPN.

Paul et al. (2009) [47]	30	65–75	DPN (15) DM without DPN (15)	Gait parameters: (1) step length; (2) duration duration of single and double support; (3) gait velocity; (4) cadence	GAITRite system.	Greater step length, lower single support time, higher double support time, slower gait velocity, and lower cadence in cognitive and motor task in DPN group compared with DM without DPN group.

Raspovic (2013) [48]	40	Not stated	DPN with foot ulcer history (10) DPN with no foot ulcer history (10) DM without DPN and ulcer history (10) Control (10)	Kinetic Kinematic	Vicon 512 Motion Analysis System. A force plate.	Gait alterations in people with clinically severe DPN and related plantar foot ulcer history.

Richardson et al. (2004) [49]	24	50–85	DPN (12) Control (12)	Gait performance: (1) step width; (2) step width variability; (3) step-width range; (4) step width-to-step length ratio; (5) step time; (6) step time variability; (7) step length; (8) step speed	Subjects placed in a safety harness that was attached by climbing rope to an overhead track and, then, walking in a SE (normal surface and lighting) and CE (irregular surface and low lighting).	DPN group demonstrated significant increases in step width, increased step-width variability, increased step-width range, increased step width-to-step length ratio, increased step time, increased step time variability, and decreased step length and speed in CE demonstrated a slower, wider-based, and more variable gait compared to SE.

Sawacha et al. (2009) [28]	67	Not stated	DPN (26) DM without DPN (21) Control (20)	Kinetic and kinematic gait	BTS motion capture system.	Altered gait was found in diabetic patients irrespective of polyneuropathy.

Savelberg et al. (2010) [50]	28	Not stated	DPN (8) DM without DPN (10) Control (10)	Gait velocity Kinematics	Body positions in the sagittal plane were recorded using a 2D digital optical recording system, which consisted of a 25 Hz interlaced digital video camera (50 frames/s).	Independent of walking speed, muscle activation differed between groups. In DPN group activation of ankle joint dorsal flexors was prolonged by 5–10% of the stride cycle.

Wuehr et al. (2014) [51]	36	>70	DPN (18) Control (18)	Walking speed Gait pattern Gait variability Temporal and spatial gait parameters	Gait analysis was performed using a 6.7 m long pressure sensitive carpet. Walking patterns were recorded during three different locomotion speeds (i.e., slow (SWS), preferred (PWS), and maximal walking speed (MWS)) with EO and during preferred walking with EC.	Alterations in the mean locomotion pattern of DPN group were mainly related to reduced walking speed. However, prolonged double support times, widened base widths, and increased gait variability during slow walking or with eyes closed appeared to be directly linked to peripheral sensory loss in patients.

Zurales et al. (2016) [52]	12	50–85	DPN (12)	Gait parameters on smooth and uneven surfaces	Optoelectronic kinematic techniques through two optoelectronic markers (infrared-emitted diodes) positioned 5 cm apart on an aluminum strip (10 cm 1.5 cm) that was bent at a 90-degree angle and inserted under the laces of each shoe at the midline.	An uneven surface is the strongest predictor of falls and injuries in older subjects with a spectrum of peripheral neurologic function.

SE: standard environment, CE: challenging environment, A/P: anterior/posterior, M/L: medial/lateral, EO: eyes open, EC: eyes closed, and BOS: base of support.

## Additional Points

There are no studies that noted on confounding factors of aging in stable posture and gait performance among DPN participants. Plus, the limited number of published studies regarding the dual cognitive or motor tasking on the gait performance in the DPN causes difficulty in reviewing the effect of DPN on the dual tasking in the gait performance. Future studies about the dual tasking on the gait performance should be considered.

## References

[1] Kiani J., Moghimbeigi A., Azizkhani H., Kosarifard S. (2013). The prevalence and associated risk factors of peripheral diabetic neuropathy in Hamedan, Iran. *Archives of Iranian Medicine*.

[2] Katulanda P., Ranasinghe P., Jayawardena R., Constantine G. R., Sheriff M. H. R., Matthews D. R. (2012). The prevalence, patterns and predictors of diabetic peripheral neuropathy in a developing country. *Diabetology and Metabolic Syndrome*.

[3] Rani P. K., Raman R., Rachapalli S., Pal S., Kulothungan V., Sharma T. (2010). Prevalence and risk factors for severity of diabetic neuropathy in type 2 diabetes mellitus. *Indian Journal of Medical Sciences*.

[4] Noor Hasimah M., Nurhanani M., Ramli M. (2010). Medical complications among type 2 diabetes mellitus patients at a general hospital in east coast Malaysia. *The International Medical Journal of Malaysia*.

[5] Zanariah H., Chandran L., Wan Mohamad W. (2008). DWP1-3 Prevalence of diabetes mellitus in Malaysia in 2006—results of the 3rd National Health and Morbidity Survey (NHMS III). *Diabetes Research and Clinical Practice*.

[6] Institute for Public Health (IPH) (2011). *National Health and Morbidity Survey 2011 (NHMS 2011). Vol. II: Non-Communicable Diseases*.

[7] Tanenberg R. J. (2009). Diabetic peripheral neuropathy: painful or painless. *Hospital Physician*.

[8] Vinik A. I. (2004). Advances in diabetes for the millennium: new treatments for diabetic neuropathies. *Medscape General Medicine*.

[9] Dixit S., Maiya A. (2014). Diabetic peripheral neuropathy and its evaluation in a clinical scenario: a review. *Journal of Postgraduate Medicine*.

[10] Gupta A., Gupta Y. (2014). *Diabetic Neuropathy: Part*.

[11] Vinik A. I., Maser R. E., Mitchell B. D., Freeman R. (2003). Diabetic autonomic neuropathy. *Diabetes Care*.

[12] El Bardawil M. M., Abd El Hamid M. M., El Sawy N. A., Megallaa M. H., El Emary W. S. (2013). Postural control and central motor pathway involvement in type 2 diabetes mellitus: dynamic posturographic and electrophysiologic studies. *Alexandria Journal of Medicine*.

[13] Dingwell J. B., Cavanagh P. R. (2001). Increased variability of continuous overground walking in neuropathic patients is only indirectly related to sensory loss. *Gait & Posture*.

[14] Dingwell J. B., Ulbrecht J. S., Boch J., Becker M. B., O'Gorman J. T., Cavanagh P. R. (1999). Neuropathic gait shows only trends towards increased variability of sagittal plane kinematics during treadmill locomotion. *Gait and Posture*.

[15] Jernigan S. D., Pohl P. S., Mahnken J. D., Kluding P. M. (2012). Diagnostic accuracy of fall risk assessment tools in people with diabetic peripheral neuropathy. *Physical Therapy*.

[16] Moher D., Liberati A., Tetzlaff J. (2009). Preferred reporting items for systematic reviews and meta-analyses: the PRISMA statement. *Annals of Internal Medicine*.

[17] Boucher P., Teasdale N., Courtemanche R., Bard C., Fleury M. (1995). Postural stability in diabetic polyneuropathy. *Diabetes Care*.

[18] Corriveau H., Prince F., Hébert R. (2000). Evaluation of postural stability in elderly with diabetic neuropathy. *Diabetes Care*.

[19] Dickstein R., Peterka R. J., Horak F. B. (2003). Effects of light fingertip touch on postural responses in subjects with diabetic neuropathy. *Journal of Neurology Neurosurgery & Psychiatry*.

[20] Dixit S., Maiya A., Shasthry B., Kumaran D., Guddattu V. (2015). Postural sway in diabetic peripheral neuropathy among Indian elderly. *Indian Journal of Medical Research*.

[21] Fahmy I. M., Ramzy G. M., Salem N. A., Ahmed G. M., Mohammed A. A. (2014). Balance disturbance in patients with diabetic sensory polyneuropathy. *Egyptian Journal of Neurology, Psychiatry and Neurosurgery*.

[22] de Souza Fortaleza A. C., Chagas E. F., Ferreira D. M. A. (2013). Postural control and functional balance in individuals with diabetic peripheral neuropathy. *Revista Brasileira de Cineantropometria & Desempenho Humano*.

[23] Fulk G. D., Robinson C. J., Mondal S., Storey C. M., Hollister A. M. (2010). The effects of diabetes and/or peripheral neuropathy in detecting short postural perturbations in mature adults. *Journal of NeuroEngineering and Rehabilitation*.

[24] Giacomini P. G., Bruno E., Monticone G. (1996). Postural rearrangement in IDDM patients with peripheral neuropathy. *Diabetes Care*.

[25] Nardone A., Grasso M., Schieppati M. (2006). Balance control in peripheral neuropathy: are patients equally unstable under static and dynamic conditions?. *Gait & Posture*.

[26] Palma F. H., Antigual D. U., Martínez S. F., Monrroy M. A., Gajardo R. E. (2013). Static balance in patients presenting diabetes mellitus type 2 with and without diabetic polyneuropathy. *Arquivos Brasileiros de Endocrinologia e Metabologia*.

[27] Salsabili H., Bahrpeyma F., Esteki A., Karimzadeh M., Ghomashchi H. (2013). Spectral characteristics of postural sway in diabetic neuropathy patients participating in balance training. *Journal of Diabetes and Metabolic Disorders*.

[28] Sawacha Z., Gabriella G., Cristoferi G., Guiotto A., Avogaro A., Cobelli C. (2009). Diabetic gait and posture abnormalities: a biomechanical investigation through three dimensional gait analysis. *Clinical Biomechanics*.

[29] Simoneau G. G., Ulbrecht J. S., Derr J. A., Becker M. B., Cavanagh P. R. (1994). Postural instability in patients with diabetic sensory neuropathy. *Diabetes Care*.

[30] Toosizadeh N., Mohler J., Armstrong D. G., Talal T. K., Najafi B. (2015). The influence of diabetic peripheral neuropathy on local postural muscle and central sensory feedback balance control. *PLoS ONE*.

[31] Turcot K., Allet L., Golay A., Hoffmeyer P., Armand S. (2009). Investigation of standing balance in diabetic patients with and without peripheral neuropathy using accelerometers. *Clinical Biomechanics*.

[32] Uccioli L., Giacomini P. G., Monticone G. (1995). Body sway in diabetic neuropathy. *Diabetes Care*.

[33] Yamamoto R., Kinoshita T., Momoki T. (2001). Postural sway and diabetic peripheral neuropathy. *Diabetes Research and Clinical Practice*.

[34] Allet L., Armand S., de Bie R. A. (2009). Gait alterations of diabetic patients while walking on different surfaces. *Gait & Posture*.

[35] Allet L., Kim H., Ashton-Miller J. A., Richardson J. K. (2012). Which lower limb frontal plane sensory and motor functions predict gait speed and efficiency on uneven surfaces in older persons with diabetic neuropathy?. *PM&R*.

[36] Brown S. J., Handsaker J. C., Bowling F. L., Maganaris C. N., Boulton A. J. M., Reeves N. D. (2014). Do patients with diabetic neuropathy use a higher proportion of their maximum strength when walking?. *Journal of Biomechanics*.

[37] Camargo M. R., Barela J. A., Nozabieli A. J. L., Mantovani A. M., Martinelli A. R., Fregonesi C. E. P. T. (2015). Balance and ankle muscle strength predict spatiotemporal gait parameters in individuals with diabetic peripheral neuropathy. *Diabetes and Metabolic Syndrome: Clinical Research and Reviews*.

[38] Chiles N. S., Phillips C. L., Volpato S. (2014). Diabetes, peripheral neuropathy, and lower-extremity function. *Journal of Diabetes and Its Complications*.

[39] Courtemanche R., Teasdale N., Boucher P., Fleury M., Lajoie Y., Bard C. (1996). Gait problems in diabetic neuropathic patients. *Archives of Physical Medicine and Rehabilitation*.

[40] de Mettelinge T. R., Delbaere K., Calders P., Gysel T., Van Den Noortgate N., Cambier D. (2013). The impact of peripheral neuropathy and cognitive decrements on gait in older adults with type 2 diabetes mellitus. *Archives of Physical Medicine and Rehabilitation*.

[41] Fortaleza A. C. D. S., Chagas E. F., Ferreira D. M. A. (2014). Gait stability in diabetic peripheral neuropathy. *Revista Brasileira de Cineantropometria & Desempenho Humano*.

[42] Katoulis E. C., Ebdon-Parry M., Lanshammar H., Vileikyte L., Kulkarni J., Boulton A. J. M. (1997). Gait abnormalities in diabetic neuropathy. *Diabetes Care*.

[43] Lalli P., Chan A., Garven A. (2013). Increased gait variability in diabetes mellitus patients with neuropathic pain. *Journal of Diabetes and Its Complications*.

[44] Manor B., Wolenski P., Li L. (2008). Faster walking speeds increase local instability among people with peripheral neuropathy. *Journal of Biomechanics*.

[45] Martinelli A. R., Mantovani A. M., Nozabieli A. J. L. (2013). Muscle strength and ankle mobility for the gait parameters in diabetic neuropathies. *Foot*.

[46] Najafi B., Khan T., Fleischer A., Wrobel J. (2013). The impact of footwear and walking distance on gait stability in diabetic patients with peripheral neuropathy. *Journal of the American Podiatric Medical Association*.

[47] Paul L., Ellis B. M., Leese G. P., McFadyen A. K., McMurray B. (2009). The effect of a cognitive or motor task on gait parameters of diabetic patients, with and without neuropathy. *Diabetic Medicine*.

[48] Raspovic A. (2013). Gait characteristics of people with diabetes-related peripheral neuropathy, with and without a history of ulceration. *Gait & Posture*.

[49] Richardson J. K., Thies S. B., DeMott T. K., Ashton-Miller J. A. (2004). A comparison of gait characteristics between older women with and without peripheral neuropathy in standard and challenging environments. *Journal of the American Geriatrics Society*.

[50] Savelberg H. H. C. M., Ilgin D., Angin S., Willems P. J. B., Schaper N. C., Meijer K. (2010). Prolonged activity of knee extensors and dorsal flexors is associated with adaptations in gait in diabetes and diabetic polyneuropathy. *Clinical Biomechanics*.

[51] Wuehr M., Schniepp R., Schlick C. (2014). Sensory loss and walking speed related factors for gait alterations in patients with peripheral neuropathy. *Gait & Posture*.

[52] Zurales K., DeMott T. K., Kim H., Allet L., Ashton-Miller J. A., Richardson J. K. (2016). Gait efficiency on an uneven surface is associated with falls and injury in older subjects with a spectrum of lower limb neuromuscular function: a prospective study. *American Journal of Physical Medicine & Rehabilitation*.

[53] Sawacha Z., Guarneri G., Cristoferi G., Guiotto A., Avogaro A., Cobelli C. (2012). Integrated kinematics-kinetics-plantar pressure data analysis: a useful tool for characterizing diabetic foot biomechanics. *Gait and Posture*.

[54] Menz H. B., Lord S. R., St George R., Fitzpatrick R. C. (2004). Walking stability and sensorimotor function in older people with diabetic peripheral neuropathy. *Archives of Physical Medicine and Rehabilitation*.

[55] Cavanagh P. R., Perry J. E., Ulbrecht J. S., Derr J. A., Pammer S. E. (1998). Neuropathic diabetic patients do not have reduced variability of plantar loading during gait. *Gait & Posture*.

[56] Wrobel J. S., Crews R. T., Connolly J. E. (2009). Clinical factors associated with a conservative gait pattern in older male veterans with diabetes. *Journal of Foot and Ankle Research*.

[57] Jáuregui-Renaud K. (2013). *Postural Balance and Peripheral Neuropathy*.

[58] Chaudhry H., Findley T., Quigley K. S. (2004). Measures of postural stability. *Journal of Rehabilitation Research and Development*.

[59] Chitra J., Shetty S. S. (2015). Screening of proprioception of ankle joint in patients with diabetic neuropathy—an observational study. *International Journal of Therapies and Rehabilitation Research*.

[60] Bonnet C. T., Lepeut M. (2011). Proximal postural control mechanisms may be exaggeratedly adopted by individuals with peripheral deficiencies: a review. *Journal of Motor Behavior*.

[61] Maurer M. S., Burcham J., Cheng H. (2005). Diabetes mellitus is associated with an increased risk of falls in elderly residents of a long-term care facility. *Journals of Gerontology—Series A Biological Sciences and Medical Sciences*.

[62] Hamada S. M., EL Debrky H. M. (2014). Monitoring of motor function affection and postural sway in patients with type 2 diabetes mellitus. *Egyptian Journal of Ear, Nose, Throat and Allied Sciences*.

[63] Bonnet C. T., Ray C. (2011). Peripheral neuropathy may not be the only fundamental reason explaining increased sway in diabetic individuals. *Clinical Biomechanics*.

[64] Petrofsky J., Macnider M., Navarro E., Lee S. (2005). Motor control and gait characteristics in people with type 1 and type 2 diabetes without sensory impairment in the foot. *Basic and Applied Myology*.

[65] Kwon O.-Y., Mueller M. J. (2001). Walking patterns used reduce forefoot plantar pressures in people width diabetic neuropathies. *Physical Therapy*.

[66] Karmakar S., Rashidian H., Chan C., Liu C., Toth C. (2014). Investigating the role of neuropathic pain relief in decreasing gait variability in diabetes mellitus patients with neuropathic pain: a randomized, double-blind crossover trial. *Journal of NeuroEngineering and Rehabilitation*.

[67] Cui X., Abduljalil A., Manor B. D., Peng C.-K., Novak V. (2014). Multi-Scale glycemic variability: a link to gray matter atrophy and cognitive decline in type 2 diabetes. *PLoS ONE*.

[68] Sommerfield A. J., Deary I. J., Frier B. M. (2004). Acute hyperglycemia alters mood state and impairs cognitive performance in people with type 2 diabetes. *Diabetes Care*.

[69] Ferguson S. C., Blane A., Perros P. (2003). Cognitive ability and brain structure in type 1 diabetes: relation to microangiopathy and preceding severe hypoglycemia. *Diabetes*.

[70] Pan X., Bai J.-J. (2014). Balance training in the intervention of fall risk in elderly with diabetic peripheral neuropathy: a review. *International Journal of Nursing Sciences*.

